# Functional Analyses of the *Bacillus velezensis* HMB26553 Genome Provide Evidence That Its Genes Are Potentially Related to the Promotion of Plant Growth and Prevention of Cotton Rhizoctonia Damping-Off

**DOI:** 10.3390/cells12091301

**Published:** 2023-05-02

**Authors:** Zhenhe Su, Gaoge Liu, Xiaomeng Liu, Shezeng Li, Xiuyun Lu, Peipei Wang, Weisong Zhao, Xiaoyun Zhang, Lihong Dong, Yuanhang Qu, Jiaqi Zhang, Shaojing Mo, Qinggang Guo, Ping Ma

**Affiliations:** Institute of Plant Protection, Hebei Academy of Agricultural and Forestry Sciences, Integrated Pest Management Innovation Center of Hebei Province, Key Laboratory of IPM on Crops in Northern Region of North China, Ministry of Agriculture and Rural Affairs of China, Baoding 071000, China; suzhenhe@haafs.org (Z.S.); liugaoge@haafs.org (G.L.); liuxiaomeng@haafs.org (X.L.); shezengli@haafs.org (S.L.); luxiuyun03@haafs.org (X.L.); peipeiwang@haafs.org (P.W.); weisongzhao@haafs.org (W.Z.); xiaoyunzhang@haafs.org (X.Z.); lihongdong@haafs.org (L.D.); quyuanhang@haafs.org (Y.Q.); zhangjiaqi@haafs.org (J.Z.); moshaojing@haafs.org (S.M.)

**Keywords:** *Bacillus velezensis*, biocontrol agent, genome mining, secondary metabolites, pathogen inhibition, cotton growth promotion

## Abstract

*Bacillus* spp. is one kind of the important representative biocontrol agents against plant diseases and promoting plant growth. In this study, the whole genomic sequence of bacterial strain HMB26553 was obtained. A phylogenetic tree based on the genome and ANI (average nucleotide identity), as well as dDDH (digital DNA–DNA hybridization), was constructed, and strain HMB26553 was identified as *Bacillus velezensis*. Fourteen biosynthetic gene clusters responsible for secondary metabolite were predicted via anti-SMASH, and six secondary metabolites were identified by UHPLC-QTOF-MS/MS (ultra-high-performance liquid chromatography coupled to quadrupole-time-of-flight tandem mass spectrometry). When the phytopathogen *Rhizoctonia solani* was treated with *B. velezensis* HMB26553, the mycelial structure changed, ROS (reactive oxygen species) accumulated, and the mitochondrial membrane potential decreased. Characteristics of strain HMB26553 were predicted and confirmed by genomic information and experiments, such as producing IAA, siderophore, extracellular enzymes and biofilm, as well as moving and promoting cotton growth. All these results suggested the mechanisms by which *B. velezensis* HMB26553 inhibits pathogen growth and promotes cotton growth, which likely provided the potential biocontrol agent to control cotton *Rhizoctonia* damping-off.

## 1. Introduction

Cotton is one of the important cash crops worldwide. In 2021, 6.5 million tones of cotton were produced in China, according to data from the Food and Agriculture Organization (FAO), and contributed significantly to China’s agricultural economy [1]. However, many diseases occurred in the cotton growth phase. Among them, cotton damping-off caused by *Rhizoctonia solani* was the important one that caused huge economic losses and was a threat to the cotton industry [2]. Therefore, how to effectively control *Rhizoctonia* cotton damping-off became a great concern. Some scientists had tried to create resistance breeding materials by transforming other exogenous genes into cotton, but little success was achieved because ideal resistance genes were lacking [3]. At present, the chemical pesticide carboxin is generally effective, but the long-term and extensive application could cause pathogens resistant to pesticides [4,5]. At the same time, chemical agents accumulated in the soil, which affected the planting of subsequent crops and polluted the environment. Due to the urgent necessity of protecting the environment and achieving green and sustainable development, biological control could provide a popular and safe alternative to traditional chemical pesticides.

The *Bacillus* genus had drawn increasing interest as a biological resource for the creation of microbial pesticides, partly because the majority of its species were environmentally friendly and helpful in controlling plant diseases [6]. *B. velezensis* was a widespread soil inhabitant that was becoming more thoroughly investigated and regarded as an effective biocontrol agent against plant diseases. The mechanisms by which *B. velezensis* controlled plant diseases included inhibiting plant pathogen growth by producing bioactive compounds, promoting plant growth by secreting plant hormones, competing with pathogens for nutrients and habitats, and inducing systemic resistance in plants [7]. Inhibition and promotion were generally important characteristics in that *B. velezensis* served as a biocontrol agent and played a key role in controlling plant diseases [8,9].

*B. velezensis* generated more than two dozen antimicrobial compounds with a remarkable range of structural types [10]. Based on biosynthetic pathways, antimicrobial compounds were divided into peptide compounds synthesized by the nonribosomal pathway, such as lipopeptides and polyketides, and small molecular compounds produced by the ribosomal pathway, such as bacteriocins [11]. Among these, lipopeptides, including fengycin surfactin and iturin families, were usually emphasized with the greatest focus [12]. Fengycin showed a strong antifungal effect against filamentous fungi by antagonizing oleic acid, phospholipids, and sterols in fungal membranes [13]. Iturin was found to affect the sterol present in the fungi’s cytoplasmic membrane [14]. Surfactin exhibited no antifungal effect. Other compounds, such as bacilysin, bacillibactin and extracellular enzymes, also potentially caused damage to phytopathogens [15,16,17]. When the fungus was under external stress, reactive oxygen species gradually accumulated and caused damage when they exceeded the normal level. At the same time, the membrane of mitochondria was also destroyed, decreasing respiration and providing energy to the fungus [18]. The capacity to promote plant growth helped advance plant healthy growth and resistance to pathogenic bacteria [19]. The most crucial growth regulators, phytohormones, such as IAA, ACC, and cytokinin, had a significant effect on direct plant growth [20]. Other factors, such as phosphorus solubilization, nitrogen fixation, and sulfur metabolism, were also key factors that indirectly promote plant growth.

A previous study reported that strain HMB26553 provided a good control effect on cotton Rhizoctonia damping-off (https://pubchem.ncbi.nlm.nih.gov/patent/CN-106939290-B (accessed on 30 December 2021)), but little was known about the detailed mechanism. In this study, some bioactive antimicrobial metabolites were predicted and identified, and the compounds, as well as strain HMB26553, resulted in a deformed structure, increased ROS and decreased mitochondrial membrane potential of *R. solani* AG-4. Plant growth-promoting traits were also assessed in vitro. The objective of this study was to demonstrate that bioactive compounds and plant growth-promoting traits of strain HMB26553 greatly contributed to preventing cotton Rhizoctonia from damping-off and promoting cotton growth.

## 2. Materials and Methods

### 2.1. Microorganisms and Culture Conditions

*B. velezensis* HMB26553 was regularly cultivated at 37 °C on the Luria Bertani medium. Strain HMB26553 was cultured in Landy broth at 30 °C and 180 rpm to produce lipopeptides and other secondary metabolites [21]. The antifungal activity test was conducted on the phytopathogen *R. solani* using a modified version of Su et al.’s approach [22]. In short, a 9-cm potato dextrose agar (PDA) plate containing a 6-mm-diameter disc of *R. solani* was infected 2 cm from the center with *B. velezensis* HMB26553 using a sterilized toothpick. After a 3-day incubation at 25 °C, the diameter of the inhibitory zone was lastly measured.

### 2.2. Genome Sequencing of Strain HMB26553

Majorbio (Shanghai, China) performed whole-genome shotgun DNA sequencing utilizing paired-end HiSeq 2000 (Illumina, San Diego, CA, USA) and long PacBio RS (Pacific Biosciences, Menlo Park, CA, USA) sequencing. A 500 bp paired-end library was produced and sequenced using an Illumina HiSeq 2000 for Illumina sequencing. Standard procedures were used to create 8–10 k insert whole–genome shotgun libraries and sequence them using a PacBio RS device for PacBio sequencing. The Celera Assembler pipeline was used to assemble the genome using SOAPdenovo (Version 2.04) and PacBioToCA using both Illumina and PacBio reads [23,24]. The NCBI Prokaryotic Genomes Automatic Annotation Pipeline (https://www.ncbi.nlm.nih.gov/genome/annotation_prok/ (accessed on 18 May 2022)) was used to annotate the genome of strain HMB26553 with GeneMark, Glimmer, and tRNAscan-SE tools [25], The Rapid Annotations by subsystems Technology (RAST) server with the seed database was used to perform functional annotation [26]. Finally, the genome and plasmids of strain HMB26553 were deposited in the GenBank database with accession numbers CP097467-CP097469 at the National Center for Biotechnology Information (NCBI; https://www.ncbi.nlm.nih.gov/ (accessed on 25 May 2022)).

### 2.3. Evolutionary Analysis

The Type Strain Genome Server (TYGS) (https://tygs.dsmz.de/ (accessed on 1 January 2022)) was used to construct a phylogenetic tree based on complete genome sequences. [27]. The average branch support values and δ statistics were used to evaluate the tree. A high delta value indicated high branch support and phylogenetic accuracy for the tree. The δ statistic value measures the tree likeness of the phylogeny [28]. The lower the delta value is, the higher the accuracy is. The ANI calculator from jspeciesws (https://jspecies.ribohost.com/jspeciesws/ (accessed on 1 January 2022)) [29] and the GGDC web servers were used to calculate the ortho ANI and dDDH similarities, respectively, between strain HMB26553 and its phylogenomic neighbors. The ANI and dDDH pairwise relatedness values were visualized by MORPHEUS (https://software.broadinstitute.org/morpheus/ (accessed on 1 January 2022)).

### 2.4. Mobile Genetic Element Analysis and Local BLAST

The mobile genetic elements, including the island gene, prophage and CRISPR–Cas of strain HMB26553, were predicted by IslandPath-DIMOB (http://www.pathogenomics.sfu.ca/islandviewer/ (accessed on 6 June 2022)), Phage Finder [30] and CRISPRFinder web tool (https://crispr.upsud.fr/Server/CRISPRfinder.php (accessed on 6 June 2022)), respectively. These genes involved in plant growth promotion were used as queries against the strain HMB26553 genome database using the local BLASTN program with an e-value of 1 × 10^−5^.

### 2.5. Prediction of Biosynthetic Gene Clusters for Secondary Metabolite

antiSMASH (http://antismash.secondarymetabolites.org (accessed on 30 March 2022)) [31] and PRISM (http://grid.adapsyn.com/prism/ (accessed on 30 March 2022)) [32] with default parameters were used to detect secondary metabolite biosynthetic gene clusters in strain HMB26553. The PKS/NRPS Analysis Website (http://nrps.igs.umaryland.edu/ (accessed on 30 March 2022)) was utilized to analyze the functional domain predictions for PKS/NRPS in the predicted gene clusters [33].

### 2.6. UHPLC-QTOF-MS/MS

The UHPLC-QTOF-MS/MS analysis conducted on a hybrid quadrupole time-of-flight tandem mass spectrometer was performed as previously described [22]. The analysis was carried out by using an HPLC equipped with LC-30AD binary pumps, a SIL-30AC autosampler, and a CTO-30AC column oven. Separation was performed using a C18 reversed-phase LC column (Shim-pack GIST 2-μm particles, 2.1 mm × 100 mm) with mobile phases A and B consisting of water and acetonitrile, respectively, both with 0.1% formic acid. The optimized linear gradient elution procedure was employed with an injection volume of 20 μL and a flow rate of 0.30 mL/min. The data were processed by using Analyst TF 1.7 software (Applied Biosystems Sciex, Toronto, ON, Canada) and PeakViewTM software 2.0 (Applied Biosystems Sciex, Toronto, ON, Canada) to interpret the mass spectral data and perform structural elucidation.

### 2.7. Assessment of Plant Growth-Promoting Traits of Strain HMB26553 In Vitro

In this section, the plant growth-promoting trait of strain HMB26553 was evaluated by testing its ability to produce extracellular enzymes, indole-3-acetic acid (IAA), and siderophores, as well as the motility in vitro.

#### 2.7.1. Extracellular Enzyme Production

Extracellular enzymes, including protease, cellulose and amylase activity, were performed as previously described [34]. In brief, 1 mL of 1 × 10^8^ cells/mL overnight culture of strain HMB26553 was spot-inoculated at the center of agar plates with the relevant substrate. Detection of protease activity was performed on LB agar plates containing 10% (*w*/*v*) skim milk powder, and the activity of the protease produced was determined by measuring the radius of the halo zone around the strain after 24 h of incubation at 30 °C. Cellulase activity was analyzed on 0.1% carboxymethylcellulose (CMC) (1 L: 5 g yeast extract, 1 g (NH_4_)_2_SO_4_, 2 mL glycerol, 0.1 g MgSO_4_, 7 g K_2_HPO_4_, 2 g KH_2_PO_4_ and 16 g agar) agar medium plates supplemented with the required components, and the circle around the colonies were photographed and quantified by ImageJ software. For amylase activity analysis, 2% soluble starch (1 L: 5 g peptone, 5 g NaCl and 16 g agar) agar medium plates were used, and transparent circles around the colonies were captured and quantified by ImageJ software after staining with 0.05% iodine solution for 5 min and washing three times with water.

#### 2.7.2. Motility

To investigate swimming and swarming motility, LB plates supplemented with 0.3% and 0.5% agar were used. A spot-inoculation was performed using 5 m of 1 × 10^8^ cells/mL overnight culture at the center of 0.3% and 0.5% agar plates, followed by incubation at 30 °C overnight. The zone of colonization on these plates was then measured to determine the motility of the cells.

#### 2.7.3. Indole-3-Acetic Acid Production

The protocol previously described was employed to conduct the indole-3-acetic acid production assay [35]. To estimate the indole-3-acetic acid (IAA) biosynthesis capacity of the isolates, the Salkowski colorimetric technique with minor modifications was used. For three days, strain HMB26553 was cultured on an LB medium that also included 5 mm of tryptophan. The supernatants were then extracted by centrifuging for 30 min at 10,000 rpm. 10 mL of orthophosphoric acid and 4 mL of Salkowski reagent were combined with 2 mL of supernatant. Following a 25-min incubation period at room temperature, the quantity of IAA was determined using the standard curve by measuring the intensity of the pink color at 530 nm.

#### 2.7.4. Siderophore Production

For the detection of siderophore production, a chromeazurol S (CAS) assay was used [36]. Briefly, strain HMB26553 was inoculated in Landy medium for 2 days at 30 °C and 180 rpm to enrich siderophores, and then the supernatant without cells was added to an equal volume of CAS and measured by a Synergy H1 microplate reader (BioTek, Hong Kong, China) at OD630. The maximum absorbance of CAS is 630 nm, and its consumption responds to the amount of siderophore production.

### 2.8. Plant Growth Promotion In Vivo

Cotton seeds (jimian11) were treated with 70% ethanol for 1 min and 2% NaClO solution for 10 min to disinfect the surface. The cotton seeds were then laid out flat on wet filter paper to begin pre-germinating at room temperature. In a greenhouse, germinating seedlings were moved to a soil that had been sterilized. In LB medium, strain HMB26553 was grown for 12 h at 30 °C and 180 rpm. Centrifugation was used to separate the cells, which were then washed in sterile water and diluted to a final concentration of 1 × 10^8^ cells/mL. 5 mL of cell suspension was added as a treatment, and the same amount of sterilized water was added as a control when seedlings were transplanted into sterilized soil. Four weeks after inoculation, the effects of HMB26553 on cotton plants were assessed.

### 2.9. Oxidative Stress and Mitochondrial Dysfunction in R. solani Induced by Strain HMB26553

A reactive oxygen species test kit (Solarbio, Beijing, China) was used to detect the amounts of intracellular reactive oxygen species. Phytopathogen was incubated at 25 °C for 3 days to obtain enough mycelium. *R. solani* mycelium was divided equally into two parts, one treated with strain HMB26553 suspension of OD600 = 1.0 for 30 min and the other treated with water as a negative control. Then, the mycelium was labeled with 10 μmol/L 2,7-dichlorodihydrofluorescein diacetate (DCFH-DA) and washed three times with phosphate buffer solution (PBS). A Synergy H1 micro-plate reader (BioTek) with an excitation wavelength of 488 nm and an emission wavelength of 525 nm was used to measure the fluorescence.

Mitochondrial membrane potential was measured by an assay kit (Solarbio, Beijing, China) with the fluorescent dye JC-10. Briefly, a disc of *R. solani* was placed in the center of a PDA plate inoculated with *B. velezensis* HMB26553 as a treatment and without inoculation as a negative control at 25 °C for 3 days. *R. solani* mycelium was harvested and incubated with JC-10 for 30 min. A Synergy H1 micro-plate reader (BioTek) with an excitation wavelength of 488 nm and an emission wavelength of 525 nm was used to measure the fluorescence.

### 2.10. Statistical Analysis

Protease, cellulose, and amylase activity areas were determined using ImageJ software (a scale was set up next to the Petri plates during photography; ImageJ utilized the scale as the known unit to compute the unknown area). Data Processing System (DPS) software (7.05) was used to process all of the data [37]. A threshold of significance of P 0.05 was used to conduct an analysis of variance (ANOVA) and a Tukey’s multiple range test to look for significant differences between the means of various treatments. A lowercase letter, in particular, signifies a significant difference.

## 3. Results

### 3.1. Genomic Features of Strain HMB26553

**Figure 1:**
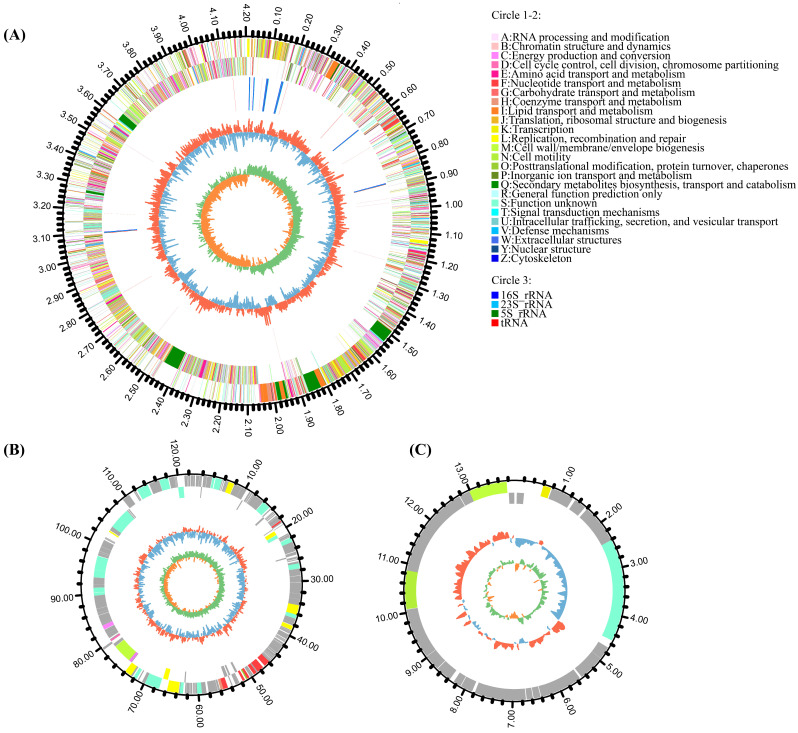
Circle genome (**A**) and plasmid (**B**,**C**) map. (**A**) The circles showed the genome and plasmids of strain HMB26553. The chromosome had four circles from 1 (outer) to 4 (inner). Circles 1–2, the COG-annotated genes (colored except grey); Circle 3, ncRNA, with different colors for different ncRNA types as shown in the figure; Circle 4, GC content; Circle 5, GC skew.

The general characteristics of strain HMB26553 genome were summarized in Figure 1 and Table 1. One circular chromosome of 4,204,437 base pairs (bp) and an average GC content of 46.40% made up the genome, along with two plasmids: plaBV1 with a length of 122,487 bp and a 35.05% GC content and plaBV2 with a length of 13,925 bp and a 43.10% GC content. Further analysis of genomic sequences identified 4714 protein-coding DNA sequences (CDS) that were distributed along both strands. One thousand five hundred seventeen genes (32.18%) out of the 4714 genes could not be annotated; the rest, 3197 genes (67.82%), had known or predicted functions in 25 different COG categories. These categories included amino acid transport and metabolism (E, 8.63%), transcription (K, 7.07%), carbohydrate transport and metabolism (G, 6.47%), translation, cell wall/membrane/envelope biogenesis (M, 5.25%), and ribosomal structure and biogenesis (J, 4.35%). Eighty-seven tRNAs and 27 rRNAs, as well as three kinds of mobile elements: 5 possible phages, 5 GIs, and 7 CRISPRs, were also found (see Table 1 and Tables S1–S3). The genome annotation of strain HMB26553 was available in NCBI under the project CP097467, and the plasmids had the accession numbers CP097468 and CP097469.

### 3.2. The Taxonomic Status of Strain HMB26553

**Figure 2:**
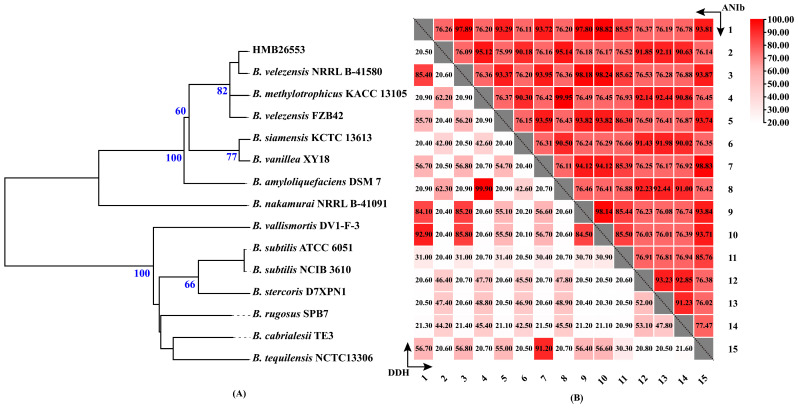
Phylogenomics-based tree and heatmap of ANI and dDDH. (**A**) Phylogenomics-based tree constructed by the type (strain) genome server TYGS (https://tygs.dsmz.de/ (accessed on 1 January 2022)). From genome sequences, Genome BLAST Distance Phylogeny (GBDP) distances were estimated. Branch lengths were scaled using Formula d5 for the GBDP distance. Branch lengths were scaled using Formula d5 for the GBDP distance. Values from 100 replications of the GBDP pseudo bootstrap were shown in the numbers above the branches. The boot-strap confidence scores were above 70%. (**B**) Heatmaps of average nucleotide identity (ANI) and dDDH analyses. For species circumscriptions, the ANI (threshold 95–96%) and dDDH (70%) values were employed. 1. HMB26553; 2. *B. stercoris* D7XPN1; 3. *B. velezensis* FZB42; 4. *B. subtilis* NCIB 3610; 5. *B. amyloliquefaciens* DSM 7; 6. *B. vallismortis* DV1-F-3; 7. *B. siamensis* KCTC 13613; 8. *B. subtilis* ATCC 6051; 9. *B. methylotrophicus* KACC 13105; 10. *B. velezensis* NRRL B-41580; 11. *B. nakamurai* NRRL B-41091; 12. *B. cabrialesii* TE3; 13. *B. rugosus* SPB7; 14. *B. tequilensis* NCTC13306; 15. *B. vanillea* XY18.

Multiple methods, including phylogenomic tree, ANI and dDDH based on the complete genome, were performed to explore the accurate taxonomy of strain HMB26553. The phylogenomic tree reconstructed on the TYGS provided evidence that strain HMB26553 was a close relative of *B. velezensis* NRRL B-41580 (Figure 2A). The average branch support value and statistics were used to evaluate the tree. The statistic value was 0.128, and the average branch support value was 61%. In addition, the dDDH and ANI values of strain HMB26553 towards *B. velezensis* NRRL B-41580 were 92.9% and 98.82%, respectively (Figure 2B), which, for prokaryotic genomic species, were over the dDDH and ANI cut-off criteria of 70% and 95%, respectively [38]. The above results confirmed that the classification of strain HMB26553 was *B. velezensis*.

### 3.3. Secondary Metabolite Biosynthetic Gene Clusters in Strain HMB26553

**Figure 3:**
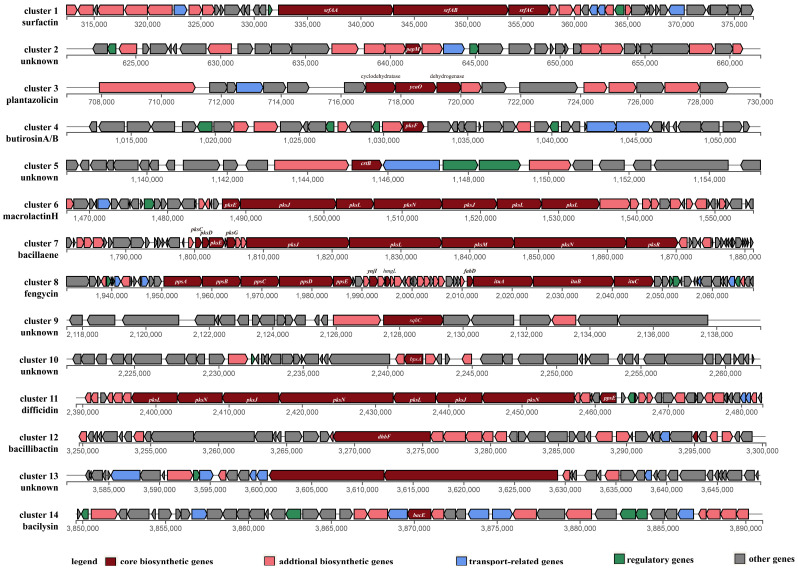
Predicted biosynthetic gene clusters of strain HMB26553 by antiSMASH. Surfactin, plantazolicin, butirosin A, macrolactin H, bacillaene, fengycin, difficidin, bacillibactin, bacilysin and other 5 unknown metabolites were predicted with core biosynthetic genes, additional biosynthetic genes, transport-related genes, regulatory genes and other genes.

The secondary metabolite biosynthetic gene clusters in the genome of strain HMB26553 were predicted using antiSMASH [31]. Two terpenes, three polyketide synthases (PKSs), one phosphonate, two hybrid NRPS-PKSs, one linear azol (in) e-containing peptide (LAP), and a gene cluster biosynthesizing bacilysin were among the 14 clusters involved in secondary metabolites that identified (Table 2). Figure 3 shows the structural makeup of the gene clusters. Core biosynthetic, additional biosynthetic, transport-related, regulatory, and other genes made up these clusters. 100% amino acid sequence homology was found between Clusters 6, 7, 8, 11, 12, and 14 and known gene clusters that produced macrolactin H, bacillaene, fengycin, difficidin, and bacilysin, respectively (Table 2). Gene Clusters 1, 3 and 4 showed 86%, 91% and 7% amino acid similarity with surfactin, plantazolicin and butirosin A synthetase gene clusters. Gene Clusters 2, 5, 9, 10 and 13 showed no similarity with the most similar known cluster in the MIBiG (Minimum Information about a Biosynthetic Gene cluster) data, among which Cluster 2 was involved in phosphonate, Clusters 5 and 9 were involved in biosythesizing terpene, Cluster 10 was T3PKS and Cluster 13 was NRPS.

**Figure 4:**
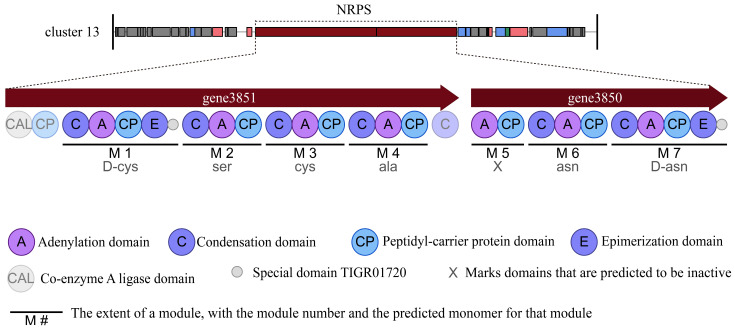
Novel biosynthetic gene cluster and potential compounds with key structures identified from strain HMB26553.

Further analysis showed that Cluster 13 was a novel NRPS gene cluster that consisted of two genes and had a total size of 28,421 bp. Two genes encoded 27 domains, which included six condensation (C) domains, seven adenylation (A) domains, eight peptidyl-carrier protein (CP) domains, two epimerization (E) domains, one coenzyme A ligase (CAL) domain and two special domains of TIGR01720 with unknown function. Most of the domains were essential components in this cluster and catalyzed the primary formation of a lipopeptide product. This cluster showed no similarity to any known biosynthetic gene clusters reported. It was predicted that Cluster 13 might biosynthesize the key structure with amino acids (D-cys)-Ser-cys-ala-asn-(D-asn) (Figure 4).

### 3.4. Secondary Metabolite Profiling Using Q-TOF MS

**Figure 5:**
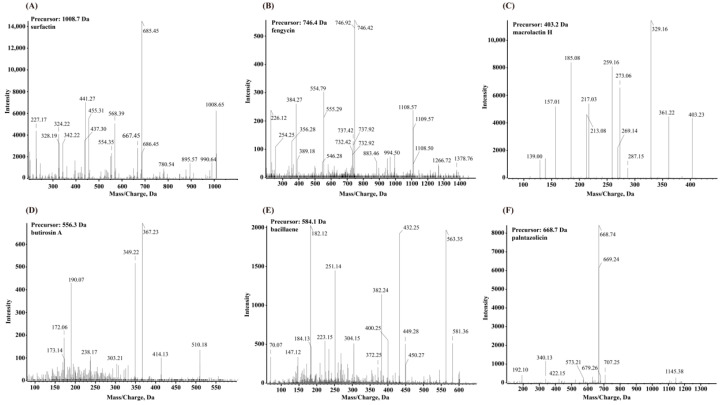
Q-TOF MS spectra obtained from the culture extract produced by strain HMB26553: protonated linear derivatives of the [M + H]^2+^ of surfactin (**A**), fengycin (**B**), macrolactin H (**C**), butirosin A (**D**), bacillaene (**E**) and plantazolicin (**F**).

The secondary metabolites produced from strain HMB26553 were identified using the UHPLC-QTOF-MS/MS method. The analyses showed that strain HMB26553 could produce various types of secondary metabolites (Figure 5). The MS/MS spectrum of the surfactin ion at *m*/*z* 1008.65 yielded one intense product ion at *m*/*z* 685.45 with an acyl chain C12. Additionally, an [M + H]^2+^ peak at *m*/*z* 746.4 for fengycin B with β-hydroxy fatty acid C16 was detected. In addition to fengycin and surfactin, four other antimicrobial compounds, macrolactin H, butirosin A, bacillaene, and plantazolicin, were extracted and detected from the fermentation broth of strain HMB26553. However, difficidin, bacillibactin and bacilysin were not detectable in the extracts by UPLC-QTOF-MS.

### 3.5. Strain HMB26553 Produced Destructive Effects on Hyphal Structure and Mitochondrial Membrane Potential

**Figure 6:**
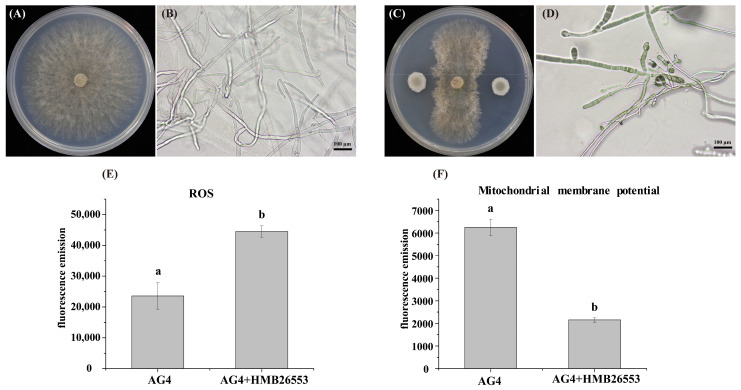
Destructive effect on the hyphal structure and mitochondrial membrane potential. Microscopic observation showing the effect of strain HMB26553 against *R. solani* (**A**,**B**) without treatment, (**C**,**D**) treatment with strain HMB26553, (**E**) increased ROS level, and (**F**) decreased mitochondrial membrane potential.

The structural changes in the morphology of fungal hyphae caused by strain HMB26553 were observed under stereomicroscopy. The untreated hyphae of *R. solani* seemed to be long, thick, and cylindrical in form. The hyphal morphology of fungal hyphae treated with strain HMB26553, on the other hand, exhibited anomalies or deformities, including curling, shrinking, distortion, and disintegration (Figure 6A–D). To clarify the specific mechanism by which strain HMB26553 produced damage to *R. solani*, the level of reactive oxygen species and mitochondrial membrane potential were examined. The results showed that fungal hyphae treated with strain HMB26553 showed lower ROS levels and mitochondrial membrane potential than that of *R. solani* fungal hyphae alone (Figure 6E,F).

### 3.6. Genomic Analysis of Plant Growth-Promoting Traits

Strain HMB26553 had a potential function in plant growth-promoting traits because its genome possessed various genes related to plant hormone production, siderophore production, extracellular enzyme production, motility, biofilm formation (Tables S5–S9), nitrogen fixation and nitrogen metabolism, phosphate solubilization and transport, sulfur metabolism and chemotaxis (Tables S10–S13). The pathway of these genes participating, as well as locus tag, gene name, and coding gene products, were summarized in the above tables. Strain HMB26553 harbored the genes of modulation of plant hormones, in which cluster *trpABCDES* and *dha*, *acdA*, *miaA*, and *nadE* contributed to producing IAA, ethylene, cytokinin, and ammonia, respectively (Table S5). Strain HMB26553 possessed genes involved in iron transport and siderophore production, including *fbpA*, *feuBC*, *fetB*, *fecCD*, *rhbE*, *yusV*, and *yfiY* (Table S6). A large number of synthesis genes related to extracellular enzymes (*nprE*, *aprX*, *amyA*, *bglAC*), motility (*swrAABC*, *pilT*, *flgBCDEGLKNM*, *flhABGPQ*, *fliCDEFGHJKLMNPQRTSYZ*, and *motAB*) and biofilm formation (*efp*, *tasA*, *crp*, *bcsBQ*, *liaG*, *welEIHGF*, and *rmlABC*) were detected in the strain HMB26553 genome (Tables S7–S9). Several other genes were responsible for nitrogen fixation and nitrogen metabolism (*gltPX*, *glnAHR*, *nadR*, *nirBD*, and *narIHJK*), phosphate solubilization and transport (*phnC*, *ispH*, *pstABCS*, *phoH*), sulfur metabolism (*cysCYKS*, *sat*, *sulP*), and chemotaxis (*cheACWV*) (Tables S10–S13).

### 3.7. Assessment and Characterization of Plant Growth-Promoting Traits of Strain HMB26553

**Figure 7:**
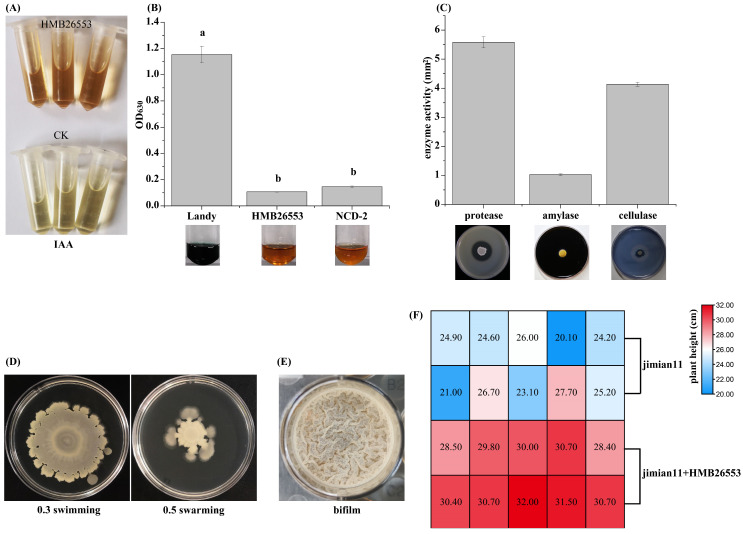
Assessment of PGPR traits in strain HMB26553. Detection of IAA production (**A**), siderophore production (**B**), extracellular enzymes including protease, amylase, and cellulase activity (**C**), motility of swimming and swarming (**D**), biofilm formation (**E**) in vitro. The cotton plant height treated with or not strain HMB26553 (**F**).

Strain HMB26553 was screened for diverse plant growth-promoting traits, such as siderophore production, IAA production, swarming and swimming motility, biofilm formation and extracellular enzyme activity, such as protease activity, cellulase activity, amylase activity. As shown in Figure 7, strain HMB26553 was positive for the above traits. In addition, the IAA production by strain HMB26553 was measured by Salkowski assay, and IAA was produced at 9.7 ± 0.3 μg/mL. Importantly, the cotton treated with strain HMB26553 showed significantly higher than that without (Figure 7F).

## 4. Discussion

The increasing cotton damping-off due to Rhizoctonia posed a serious threat to cotton production and caused huge economic losses to cotton farmers. Due to the urgent necessity of protecting the environment and achieving green and sustainable development, biological control, especially *Bacillus,* was likely a popular and safe alternative to traditional chemical pesticides. A previous study confirmed that strain HMB26553 had a good control effect on cotton Rhizoctonia damping-off (https://pubchem.ncbi.nlm.nih.gov/patent/CN-106939290-B (accessed on 30 December 2021)). However, little was known about the detailed mechanism. In this study, some bioactive antimicrobial metabolites were predicted and identified. The strain of HMB26553 and its metabolites resulted in a deformed structure, increasing ROS and decreasing mitochondrial membrane potential in the phytopathogen *R. solani* hyphae. Plant growth-promoting traits were also assessed in vitro. This study’s goal was to provide clarification that both bioactive secondary metabolites and plant growth-promoting traits of strain HMB26553 greatly contributed to preventing Rhizoctonia damping-off in cotton and promoting cotton growth.

A previous study identified strain HMB26553 as *B. subtilis* by 16S rDNA sequence phylogenetic tree. Generally, the resolution of the 16S rDNA phylogenic tree was sometimes difficult to distinguish itself from the same genus strain [34]. MLSA, genome evolutionary tree analysis, ANI analysis, and dDDH are currently the most widely used techniques for analyzing taxonomic status. However, MLSA offered less resolution in the taxonomic study of closely related bacteria than these other techniques [39]. This work employed ANI, dDDH, and genome evolutionary tree analysis in conjunction to precisely determine the taxonomic position of strain HMB26553 [39]. It was confirmed that strain HMB26553 was *B. velezensis* but not *B. subtilis*.

*Bacillus* species were important biocontrol agents in agriculture, and Wang et al. reviewed that the *B. amyloliquefaciens* Group, *B. amyloliquefaciens*, *B. velezensis*, *B. nakamurai*, and *B. siamensis* had biocontrol ability for the management of fungal postharvest diseases [40]. Some excellent *Bacillus* spp. inhibited the mycelial growth of pathogens and the germination of conidia [41]. *B. velezensis* TSA32–1 showed antagonistic effects towards various plant pathogens [42], and *B. mycoides* caused significant inhibition of *Botrytis cinerea* conidial germination, in which the conidia were broken and destroyed [43]. Many kinds of active metabolites, including bacteriocins, antibiotics, and extracellular enzymes produced by *Bacillus*, contributed greatly to the suppression of phytopathogens. In this study, 14 secondary metabolite gene clusters were predicted by genome mining, and some were identified using UHPLC-QTOF-MS/MS. Strain HMB26553 might generate at least 14 different types of secondary metabolites in total, including surfactin, plantazolicin, butirosin A, macrolactin H, bacillaene, fengycin, difficidin, bacillibactin, bacilysin, one terpene, one phosphonate and two unknown products with T3PKS and NRPS. Among the important metabolites synthesized by *Bacillus* species, lipopeptides were usually emphasized, with the greatest focus on the fengycin, surfactin and iturin families [12]. Fengycin showed potent antifungal activity against filamentous fungi by antagonizing sterols, phospholipids, and oleic acid in fungal membranes [13]. Surfactin exhibited antibacterial, antiviral, antitumor and hemolytic action [44]. Plantazolicin damages the bacterial bilayer by entering the bacteria envelope and interfering with the membrane potential [45]. Butirosin A was a member of the second class of aminoglycoside antibiotics possessing a 2-deoxystreptamine aminocyclitol core [46]. Macrolactin H showed potent antibacterial activity against *Escherichia coli*, *B. subtilis* and *Staphylococcus aureus* [47]. Difficidin was an effective antibacterial agent that rapidly inhibited protein synthesis and possibly damaged cell membranes [48]. Bacilysin exhibited antimicrobial activities against bacteria, *Candida albicans* and algal cells [49,50]. Bacillaene prevented bacterial growth by inhibiting the production of prokaryotic proteins [51]. Bacillibactin acted as a siderophore to compete for irons with ambient microbes, in particular when there was an iron deficit [52]. However, only surfactin, plantazolicin, butirosin A, bacillaene, fengycin, and bacilysin were successfully detected from the extract of strain HMB26553 by UHPLC-QTOF-MS/MS. Diddicidin, bacillibactin and macrolactin H remained undetectable regardless of the three secondary metabolite gene synthetic clusters showing 100% similarity with known clusters. The low expression level of their biosynthetic gene clusters under the experimental conditions was most likely the cause of their undetectability. Interestingly, the siderophore was detected by CAS methods, but UHPLC–MS/MS did not identify bacillibactin. Other types of siderophores, but not bacillibactin, might be the main siderophores in strain HMB26553. Among these bioactive metabolites produced by strain HMB26553, only fengycin and bacilysin likely contributed to inhibiting the growth of *R. solani*. Other metabolites, such as surfactin, butirosin A, bacillaene, and plantazolicin, seemingly suppressed bacteria, which might cooperate with fengycin and bacilysin to defeat *R solani* and accelerate fungal death. Butirosin A was more frequently produced in *B. circulans* [53], and this was first reported in species *B. velezensis*. New NRP with an unknown structure of amino acids (D-cys)-Ser-cys-ala-asn-(D-asn), new metabolites from T3PKS and a terpene derivative might also play key roles in inhibiting *R solani*.

According to reports, several antibiotics caused pathogenic fungi to undergo excessive ROS-induced oxidative stress [54]. ROS accumulation and high levels could trigger harmful stress reactions in fungi, which would affect the physiological condition of the cell and normally functioning mitochondria and result in oxidative death [55]. On the other hand, *Aspergillus fumigatus*’s defective mitochondria lessen fitness, metabolic alterations, and vulnerability to oxidative stress. Inactivation of the alternative mito oxidase resulted in increased ROS and easier death of *A. fumigatus* [56]. At the same time, bioactive metabolites specifically bind to the cell wall, cell membrane, and other targets of fungi, which harms the mycelium structure and function. Moreover, disrupted mitochondria could not supply enough energy to fulfill normal physiology and metabolism in fungi. All the factors resulted in the death of phytopathogens.

The probiotic effect of *Bacillus* was important for plant growth and its ability to promote healthy growth and resistance to pathogenic bacteria [57]. Plant growth-promoting-related genes were analyzed based on the whole genome sequence of strain HMB26553, and phytohormone genes for the synthesis of IAA, ACC, and cytokinin, genes for the synthesis of bacillibactin, extracellular enzymes for degradation genes, motility genes, and biofilm genes were found in strain HMB26553. The most significant growth regulators, phytohormones, were well recognized for having a significant influence on plant metabolism and for being essential in stimulating the defensive response systems of plants to stresses [58]. Siderophores aid in iron acquisition from insoluble hydroxide and soluble forms to starve other living organisms in the environment. Extracellular enzymes produced by the microbial community in the rhizosphere were responsible for the degradation of various components of fungal phytopathogens. Increasing evidence indicated that crucial bioprotection processes might be driven by the spatial organization of microbial populations on crop surfaces [59]. In addition, genes for phosphorus solubilization, nitrogen fixation, and sulfur metabolism were also identified in strain HMB26553. In conclusion, the functions performed by the above genes contributed to making strain HMB26553 an excellent plant growth-promoting strain. To demonstrate the probiotic traits of strain HMB26553, various properties were evaluated separately in vitro. The results showed that strain HMB26553 could produce IAA in fixed concentrations, and its continuous production should achieve the effect of promoting plant growth. Other forms of siderophores but not bacillibaction might cause phytopathogen *R. solani* iron starvation, especially in iron deficiency, which would limit the growth of the fungus. Extracellular enzymes, including protease, amylase and cellulase, were important cell wall-degrading enzymes that could degrade the cell wall and promote fungal lysis and death. Although siderophores and extracellular enzymes of strain HMB26553 did not directly promote plant growth, their ability to limit fungal growth and lyse fungi provided a healthy environment for plants, while fungal lysates could be used as nutrients for uptake by plants. Therefore, to some extent, siderophores and extracellular enzymes indirectly promoted the growth of plants. Furthermore, cotton treated with strain HMB26553 in a greenhouse showed an increased plant height, proving that strain HMB26553 was a growth-promoting strain.

The above results might lead to the development of a new biological control agent against the phytopathogen *R. solani,* as well as plant growth-promoting agents for cotton and improve our knowledge of the detailed mechanism.

## 5. Conclusions

In this study, the full genome sequence of *B. velezensis* HMB26553 was obtained. Strain HMB26553 was confirmed to be *B. velezensis* based on the phylogenomic tree, ANI and dDDH. Secondary metabolite biosynthetic gene clusters were mined, and bioactive compounds were identified by UHPLC-QTOF-MS/MS. Additionally, changes in mycelial structure, accumulation of ROS and decreased mitochondrial membrane potential were observed in the phytopathogen *R. solani* when treated with *B. velezensis* HMB26553. Plant growth-promoting traits, such as IAA production, siderophore production, extracellular enzyme production, motility, biofilm formation and cotton growth promotion in greenhouses, were confirmed by experiments. Our findings contribute to the understanding of the diversity and complexity of secondary metabolism in *B. velezensis* HMB26553, which is useful for its potential biocontrol agent to fight against phytopathogen in agriculture.

## Figures and Tables

**Table 1:** Genomic features of strain HMB26553.

Genetic Element	Assembly Size (bp)	G + C Content (%)	No. of CDS	No. of rRNA	No. of tRNA	Accession No.
Chromosome	4,204,437	46.40	4487	27	87	CP097467
plaBV1	12,2487	35.05	201	0	0	CP097468
plaBV2	13,925	43.10	26	0	0	CP097469

**Table 2:** Predicted biosynthetic gene clusters of strain HMB26553.

Region	Type	From	To	Most Similar Known Cluster	Similarity
Region 1	NRPS	312,439	376,782	Surfactin	86%
Region 2	phosphonate	620,932	661,819	-	-
Region 3	LAP	707,835	730,017	Plantazolicin	91%
Region 4	PKS-like	1,011,172	1,052,416	Butirosin A	7%
Region 5	Terpene	1,138,005	1,155,279		
Region 6	TransAT-PKS	1,467,111	1,555,235	Macrolactin H	100%
Region 7	TransAT-PKS, T3PKS, NRPS	1,781,428	1,890,245	Bacillaene	100%
Region 8	NRPS, betalactone, TransAT-PKS	1,931,028	2,068,213	Fengycin	100%
Region 9	terpene	2,117,504	2,139,387	-	-
Region 10	T3PKS	2,220,991	2,262,091	-	-
Region 11	TransAT-PKS	2,376,729	2,482,902	Difficidin	100%
Region 12	NRPS, bacteriocin	3,429,746	3,300,255	Bacillibactin	100%
Region 13	NRPS	3,580,856	3,649,276	-	-
Region 14	other	3,849,599	3,891,017	Bacilysin	100%

## Data Availability

The complete genome and plasmid sequences of *B. velezensis* HMB26553 were deposited in the NCBI genome database under accession numbers CP097467-CP097469.

## Supplementary Materials

The following supporting information can be downloaded at https://www.mdpi.com/article/10.3390/cells12091301/s1: Table S1: Characterization of mobile element prophages in the strain HMB26553 genome and plasmid; Table S2: Characterization of mobile element gene islands in the strain HMB26553 genome and plasmid; Table S3: Characterization of mobile element Crisprs in the strain HMB26553 genome and plasmid; Table S4: The genome of GenBank accession No used in this study; Table S5: Genes involved in the modulation of plant hormones; Table S6: Genes involved in iron transport and siderophore production; Table S7: Genes involved in extracellular enzymes; Table S8: Genes involved in motility; Table S9: Genes involved in biofilm formation; Table S10: Genes involved in nitrogen fixation and nitrogen metabolism; Table S11: Genes involved in phosphate solubilization and transport; Table S12: Genes involved in sulfur metabolism; Table S13: Genes involved in chemotaxis.

## Abbreviations

ANI: average nucleotide identity; dDDH: digital DNA–DNA hybridization; UHPLC-QTOF-MS/MS: ultra-high-performance liquid chromatography coupled to quadrupole-time-of-flight tandem mass spectrometry; ROS: reactive oxygen species; IAA: indole-3-acetic acid; ACC: 1-aminocyclopropane-1-carboxylate; CMC: carboxy-methylcellulose; CAS: a chromeazurol S; GBDP: genome BLAST distance phylogeny; NRPS: nonribosomal peptide synthase; PKS: polyketide synthase; LAP: linear azol (in) e-containing peptide; MIBiG: minimum information about a biosynthetic gene cluster; C domain: condensation domain; A domain: adenylation domain; CP: peptidyl-carrier protein domain; E domain: epimerization domain; CAL domain: coenzyme A ligase domain; MLSA: multilocus sequence analysis.
