# Supplementary material for: Functional Analyses of the Bacillus velezensis HMB26553 Genome Provide Evidence That Its Genes Are Potentially Related to the Promotion of Plant Growth and Prevention of Cotton Rhizoctonia Damping-Off
Source: Cells. 2023 May 2;12(9):1301. doi: 10.3390/cells12091301 (PMC10177454; doi:10.3390/cells12091301)
Supplement: Supplementary file 1 [file cells-12-01301-s001.zip › cells-2317261-supplementary.pdf]

Functional Analyses of the *Bacillus velezensis* HMB26553 Genome Provide Evidence That its Genes are Potentially Related to the Promotion of Plant Growth and Prevention of Cotton Rhizoctonia Damping-Off

Table S1 Characterization of mobile element prophages in HMB26553 genome and plasmid

Location	Ph No.	Ph Start	Ph End	Length (bp)	CDS No.	Possible Phage	GC(%)
Chromosome	Ph01	5772	88890	83119	146	Bacillus_phage_SPBc2_NC_001884	35.5
Chromosome	Ph02	898486	946156	47671	64	Bacillus_phage_SPBc2_NC_001884	48.81
Chromosome	Ph03	1288332	1320760	32429	45	Streptococcus_phage_EJ-1_NC_005294	46.86
Chromosome	Ph04	2807911	2853628	45718	77	Listeria_phage_B054_NC_009813	43.37
Chromosome	Ph05	3110944	3187878	76935	101	Bacillus_phage_SPBc2_NC_001884	47.44

Table S2 Characterization of mobile element gene islands in HMB26553 genome and plasmid

Location	GI No.	Island Start	Island End	Length(bp)	CDS No.	GC(%)
Chromosome	GI01	2181453	2211626	30173	34	36.10
Chromosome	GI02	2808272	2828726	20454	28	42.11
Chromosome	GI03	3114033	3189050	75017	99	47.64
Chromosome	GI04	874326	945805	71479	89	48.66
plaBV1	GI05	17275	20260	2985	8	37.82

Table S3 Characterization of mobile element Crisprs in HMB26553 genome and plasmid

Location	Crisprs_ No.	Crisprs_start	Crisprs_end	DR_number	DR_Average_length	SPA_Average_length
Chromosome	CRISPR1	454669	454942	5	34	26
Chromosome	CRISPR2	754240	754886	9	45	30
Chromosome	CRISPR3	1307821	1308584	10	35	46
Chromosome	CRISPR4	1352905	1352986	2	28	26
Chromosome	CRISPR5	1355208	1355572	6	23	45
Chromosome	CRISPR6	2774436	2774797	5	32	50
Chromosome	CRISPR7	3727004	3727254	4	26	49

Table S4 The genome of GenBank accession No used in this study.

strains	GenBank accession No.	strains	GenBank accession No.
HMB26553	CP097467	*B. vanillea* XY18	GCF_000966575
B. stercoris D7XPN1	GCF_000738015	*B. methylotrophicus* KACC 13105	GCF_000960265
B. velezensis FZB42	GCF_000015785	*B. velezensis* NRRL B-41580	GCF_001461825
B. subtilis NCIB 3610	GCF_000186085	*B. nakamurai* NRRL B-41091	GCF_001584325
B. amyloliquefaciens DSM 7	GCF_000196735	*B. cabrialesii* TE3	GCF_004124315
B. vallismortis DV1-F-3	GCF_000245315	*B. rugosus* SPB7	GCF_011745685
B. siamensis KCTC 13613	GCF_000262045	*B. tequilensis* NCTC13306	GCF_900445435
B. subtilis ATCC 6051	GCF_006088795		

Table S5. Genes involved in the modulation of plant hormones.

Pathway	Gene	Product	Locus Tag
IAA production (L-tryptophan pathway)	*trpA*	tryptophan synthase subunit alpha TrpA	gene2390
	trpB	tryptophan synthase subunit beta TrpB	gene2391
	trpC	indole-3-glycerol phosphate synthase TrpC	gene2393
	trpD	anthranilate phosphoribosyltransferase	gene2394
	trpE	anthranilate synthase component I	gene2395
IAA production IPA pathway	*trpS*	Tryptophan-tRNA ligase	gene1315
	dhaS	aldehyde dehydrogenase DhaS	gene2186
	dha	aldehyde dehydrogenase	gene3130 gene4357
	ywkB	auxin efflux carrier	gene4057
ACC catabolism (ethylene)	*acdA*	acyl-CoA dehydrogenase AcdA	gene0493 gene2060 gene2564
cytokinin biosynthesis and transformation	*miaA*	(adenosine(37)-N6)-dimethylallyltransferase MiaA	gene1971
ammonia production	*nadE*	ammonia-dependent NAD(+) synthetase	gene0328

Table S6. Genes involved in iron transport and siderophore production.

Pathway	Gene	Product	Locus Tag
Iron(III) transport	*fbpA*	fur-regulated basic protein FbpA	gene1537
Iron(II) transport	*fetB*	iron export ABC transporter permease subunit FetB	gene1420
	feuC	Iron-uptake system permease protein FeuC	gene0202
	feuB	ferrichrome ABC transporter permease	gene0203
	ND	iron-uptake system-binding protein	gene0204
	fecCD	iron complex transport system permease protein	gene0406
	fecCD	iron chelate uptake ABC transporter family permease subunit	gene0407
	ND	iron ABC transporter ATP-binding protein	gene0408
	ND	iron ABC transporter substrate-binding protein	gene0409
	ND	iron-dicitrate ABC transporter substrate-binding protein	gene1219
Siderophore transport	*rhbE*	rhizobactin siderophore biosynthesis protein	gene1168
	ND	2,3-dihydroxybenzoate-AMP ligase	gene3487
	ND	2,3-dihydro-2,3-dihydroxybenzoate dehydrogenase	gene3489
	yusV	iron-enterobactin transporter ATP-binding protein	gene3599
	yfiY	iron-hydroxamate ABC transporter substrate-binding protein	gene4230

Key: ND—not determined.

Table S7. Genes involved in extracellular enzymes.

Pathway	Gene	Product	Locus Tag
protease	*yabG*	sporulation-specific protease YabG	gene0056
	ftsH	ATP-dependent zinc metalloprotease FtsH	gene0085
	clpC	ATP-dependent Clp protease ATP-binding subunit ClpC	gene0117
	ydcA	rhomboid family intramembrane serine protease YdcA	gene0504
	ydiL	CAAX prenyl protease YdiL	gene0672
	ND	CAAX protease	gene0787
	ywpE	cysteine protease YwpE	gene0822
	ND	serine protease	gene0910
	clpB	Clp protease ClpB	gene1022
	yhfN	metalloprotease YhfN	gene1215
	ND	Alkaline protease	gene1216
	yjaZ	Zn-dependent protease YjaZ	gene1308
	xkdF	phage serine protease XkdF	gene1453
	ND	serine protease	gene1491
	ND	serine protease	gene1524
	htpX	protease HtpX	gene1549
	clpX	Clp protease ClpX	gene1575
	ampS	thermophilic metalloprotease (M29) AmpS	gene1653
	nprE	neutral protease	gene1692
	ylbL	Lon-like protease YlbL	gene1727
	ND	RP-I protease	gene1752
	clpQ	ATP-dependent protease subunit ClpQ	gene1842
	hslU	ATP-dependent protease ATP-binding subunit HslU	gene1843
	rseP	RIP metalloprotease RseP	gene1884
	ymxG	zinc protease YmxG	gene1898
	tepA	Clp protease TepA	gene1906
	ymfF	metalloprotease YmfF	gene1913
	ymfH	zinc protease YmfH	gene1914
	aprX	alkaline serine protease AprX	gene1961
	ctpA	carboxy-terminal processing protease CtpA	gene2223
	prsW	protease PrsW	gene2422
	ypbD	caax amino terminal protease	gene2429
	ND	sporulation factor IV B protease	gene2581
	yqeZ	CLP_protease YqeZ	gene2703
	gpr	germination protease gpr	gene2718
	yrrO	protease YrrO	gene2807
	yrrN	protease YrrN	gene2808
	Prp	cysteine protease Prp	gene2869
	La	Lon protease 1	gene2897
	lonB	ATP-dependent protease LonB	gene2898
	clpX	ATP-dependent Clp protease ATP-binding subunit ClpX	gene2899
	sppA	CLP_protease SppA	gene3113
	yraA	cysteine protease YraA	gene3138
	ND	ATP-dependent Clp protease proteolytic subunit	gene3322
	pfpI	protease PfpI	gene3401
	yugP	neutral zinc metallopeptidase YugP	gene3413
	htrB	serine protease HtrB	gene3604
	ND	reprolysin (M12B) family zinc metalloprotease	gene3662
	clpP	ATP-dependent Clp protease proteolytic subunit clpP	gene3782
	minJ	serine protease MinJ	gene3853
	ctpB	carboxy-terminal processing protease CtpB	gene3855
	ywhC	zinc metalloprotease YwhC	gene4103

	vpr	minor extracellular protease vpr	gene4172
	yjfC	Zn-dependent protease YjfC	gene4200
	epr	minor extracellular protease Epr	gene4204
	htpX	protease HtpX	gene4364
	yyxA	serine protease	gene4405
	epr	minor extracellular protease Epr	gene4456
	yyaK	caax amino terminal protease	gene4462
	yyaC	spore protease YyaC	gene4477
amylase	*amyA*	alpha-glucosidase	gene0301<br>gene0320<br>gene3411<br>gene3784<br>gene3790
cellulase	*ND*	alpha-phosphotrehalase	gene0843
	ND	arabinogalactan endo-1,4-beta-galactosidase	gene1397
	ND	endo-1,4-beta-glucanase	gene2091
	bglC	aryl-phospho-beta-D-glucosidase BglC	gene0354
	bglA	aryl-phospho-beta-D-glucosidase BglA	gene2221
	ND	6-phospho-beta-galactosidase	gene1403
	ND	glycosyl hydrolase	gene2219
	celF	6-phospho-beta-glucosidase CelF	gene4220
	bglA	6-phospho-beta-glucosidase BglA	gene4236

Table S8. Genes involved in motility.

Pathway	Gene	Product	Locus Tag
Motility	*swrC*	swarming motility protein SwrC	gene0745
	pilT	twitching motility protein PilT	gene0749
	yjfB	putative motility protein YjfB	gene1411
	motB	flagellar motor protein MotB	gene1572
	motA	flagellar motor protein MotA	gene1573
	flgB	flagellar biosynthesis protein FlgB	gene1845
	flgC	flagellar basal body rod protein FlgC	gene1846
	fliE	flagellar hook-basal body complex protein FliE	gene1847
	fliF	flagellar M-ring protein FliF	gene1848
	fliG	flagellar motor switch protein FliG	gene1849
	fliH	flagellar assembly protein FliH	gene1850
	ND	Flagellum-specific ATP synthase	gene1851
	fliJ	flagellar biosynthesis chaperone FliJ	gene1852
	fliK	flagellar hook-length control protein FliK	gene1854
	flgD	flagellar basal body rod modification protein FlgD	gene1855
	flgG	flagellar basal body rod protein FlgG	gene1856
	fliL	flagellar basal body-associated protein FliL	gene1858
	fliM	flagellar motor switch protein FliM	gene1859
	fliY	flagellar motor switch phosphatase FliY	gene1860
	fliZ	flagellar biosynthesis protein FliZ	gene1862
	fliP	flagellar biosynthetic protein FliP	gene1863
	fliQ	flagellar export apparatus protein FliQ	gene1864
	fliR	flagellar biosynthesis protein FliR	gene1865
	flhB	flagellar biosynthesis protein FlhB	gene1865
	flhA	flagellar biosynthesis protein FlhA	gene1867
	flhF	flagellar biosynthesis regulator FlhF	gene1868
	swrB	swarming motility protein SwrB	gene1876
	ypfA	increasing levels of c-di-GMP lead to decreased motility YpfA	gene2419
	swrAA	swarming motility protein SwrAA	gene3854
	fliT	flagellar protein FliT	gene3865

fliS	flagellar protein FliS	gene3866
fliD	flagellar hook-associated protein	gene3867
fliC	Flagellin fliC	gene3868
fliW	flagellar assembly protein FliW	gene3870
flgL	flagellar hook-associated protein FlgL	gene3872
flgK	flagellar hook-associated protein FlgK	gene3873
flgN	flagellar protein FlgN	gene3874
flgM	flagellar biosynthesis anti-sigma factor FlgM	gene3875
flhP	flagellar hook-basal body protein FlhP	gene3985
flhO	flagellar basal body rod protein subunit C	gene3986

Table S9. Genes involved in biofilm formation.

Pathway	Gene	Product	Locus Tag
Biofilm formation	*efp*	elongation Factor P	gene2604
	tasA	camelysin metallo-endopeptidase tasA	gene2620
	hfq	RNA chaperone Hfq	gene1972
	crp	Crp/Fnr family transcriptional regulator	gene1522<br>gene4085
EPS	*wzd*	Auxiliary protein for polysaccharide export and chain length determination	gene3972
	wze	Autophosphorylating tyrosine-protein kinase	gene3757
	wzy	Repeat unit polymerase	gene3458
	wzx	Repeat unit transporter	gene3184
	welI	Glycosyltransferase	gene3753
	welH	Glycosyltransferase	gene3887
	welG	Glycosyltransferase	gene3155
	welF	Glycosyltransferase	gene2370
	welE	Undecaprenyl-phosphate galactose phosphotransferase; priming glycosyltransferase	gene3893
	rmlA	dTDP-glucose pyrophosphorylase	gene4147
	rmlC	dTDP-4-dehydro-rhamnose-3,5-epimerase	gene4144
	rmlB	dTDP-d-glucose-4,6-dehydratase	gene4146
	wzr	Transcriptional regulator of polysaccharide biosynthesis	gene3897
	wzb	Phosphotyrosine protein phosphatase	gene3970
Adhesin	*liaG*	Adhesion LiaG	gene3618
	yvlB	Adhesion YvlB	gene3835
	liaG	Adhesion liaG	gene4141
cellulose synthase	*bcsB*	cellulose synthase bcsB	gene0458
	BcsQ	cellulose synthase bcsQ	gene1869<br>gene2873<br>gene3971<br>gene4479

Table S10. Genes involved in nitrogen fixation and nitrogen metabolism.

Pathway	Gene	Product	Locus Tag
Nitrogen fixation	*nif3-like*	nitrogen fixation protein Nif 3-like	gene2679
Nitrogen metabolism	*gltP*	glutamate/aspartate: proton symporter GltP	gene1208<br>gene0268
	gltX	Glutamate-tRNA ligase	gene0123
	glnR	transcriptional repressor GlnR	gene1985
	glnA	type I glutamate-ammonia ligase/glutamine	gene1986
	glnH	ABC transporter substrate-binding protein GlnH	gene2818
Nitrogen regulation	*nadR*	transcription repressor NadR	gene2863
Dissimilatory nitrate	*nirB*	nitrite reductase (NADH) large subunit	gene0346
	nirD	nitrate reductase (NADH) small subunit	gene0345

reduction	*narI*	respiratory nitrate reductase subunit gamma	gene4078
	narH	nitrate reductase subunit beta/nitrate reductase	gene4080
	narJ	molybdenum cofactor assembly chaperone	gene4079
	nar	nitrate reductase subunit alpha	gene4081
	nark	nitrate transporter	gene0349
Ammonia assimilation	*gltX*	glutamate-tRNA ligase	gene0123
	gltP	glutamate/aspartate: proton symporter GltP	gene1208 gene0268

Table S11. Genes involved in phosphate solubilization and transport.

Pathway	Gene	Product	Locus Tag
Degradation of phosphonates	*phnC*	phosphonate transport system ATP-binding	gene2549
	ispH	4-hydroxy-3-methylbut-2-enyl diphosphate reductase	gene2678
	pstA	phosphate ABC transporter, permease protein PstA	gene2658
	pstC	phosphate ABC transporter permease subunit PstC	gene2659
	pstS	phosphate transport system permease protein	gene2660
	pstB	phosphate import ATP-binding protein pstB	gene3698 gene2656 gene2657
	gltP	proton glutamate symport protein	gene1208 gene0268
	phoH	phosphate starvation protein PhoH	gene2698

Table S12. Genes involved in sulfur metabolism.

Pathway	Gene	Product	Locus Tag
Sulfate transport	*cysC*	adenylyl-sulfate kinase	gene1784
	sat	sulfate adenylyltransferase	gene1783
	cysT	sulfate transport system permease protein	gene0767
	cysK	cysteine synthase A	gene3176
	cysS	cysteine--tRNA ligase	gene0126
	sulP	sulfate permease	gene0199 gene1782 gene3794

Table S13. Genes involved in chemotaxis.

Pathway	Gene	Product	Locus Tag
Chemotaxis	*cheV*	chemotaxis protein CheV	gene1613
	ND	chemotaxis response regulator	gene1870
	cheA	chemotactic two-component sensor histidine kinase	gene1871
	cheW	purine-binding hemotaxis protein CheW	gene1872
	cheC	CheY-P-specific phosphatase CheC	gene1873
	ND	chemoreceptor glutamine deamidase CheD	gene1874
	ND	chemotaxis protein	gene0798
	ND	chemotaxis protein	gene1606
	che	methyl-accepting chemotaxis protein	gene3402 gene3403 gene3404 gene3405 gene1224
